# Scoring and psychometric validation of the Perception of Anticoagulant Treatment Questionnaire (PACT-Q^©^)

**DOI:** 10.1186/1477-7525-7-30

**Published:** 2009-04-07

**Authors:** MH Prins, I Guillemin, H Gilet, S Gabriel, B Essers, G Raskob, SR Kahn

**Affiliations:** 1 The Department of Epidemiology, Care and Public Health Research Institutes, University of Maastricht, Maastricht, the Netherlands; 2 Department of Clinical Epidemiology and Medical Technology Assessment, Academic Hospital, Maastricht, the Netherlands; 3 Mapi Values, Lyon, France; 4 Sanofi-Aventis, Paris, France; 5 Department of Clinical Epidemiology and Medical Technology Assessment, University Hospital Maastricht, the Netherlands; 6 Department of Medicine, University of Oklahoma Health Sciences Center, Oklahoma City, USA; 7 Department of Medicine, McGill University Division of Internal Medicine, and Center for Clinical Epidemiology & Community Studies Jewish General Hospital, Montreal, Canada

## Abstract

**Background:**

The 'Perception of Anti-Coagulant Treatment Questionnaire' (PACT-Q) was developed to assess patients' expectations of, and satisfaction with their anticoagulant treatment. This questionnaire needs to be finalised and psychometrically validated.

**Methods:**

The PACT-Q was included in the United States, the Netherlands and France into three phase III multinational clinical trials conducted to evaluate efficacy and safety of a new long-acting anticoagulant drug (idraparinux) compared to vitamin K antagonist (VKA). PACT-Q was administered to patients with deep venous thrombosis (DVT), atrial fibrillation (AF) or pulmonary embolism (PE) at Day 1, to assess patients' expectations, and at 3 and 6 months to assess patients' satisfaction and treatment convenience and burden. The final structure of the PACT-Q (Principal Component Analysis – PCA – with Varimax Rotation) was first determined and its psychometric properties were then measured with validity of the structure (Multitrait analysis), internal consistency reliability (Cronbach's alpha coefficients) and known-group validity.

**Results:**

PCA and multitrait analyses showed the multidimensionality of the "Treatment Expectations" dimension, comprising 7 items that had to be scored independently. The "Convenience" and "Burden of Disease and Treatment" dimensions of the hypothesised original structure of the questionnaire were combined, thus resulting in 13 items grouped into the single dimension "Convenience". The "Anticoagulant Treatment Satisfaction" dimension remained unchanged and included 7 items. All items of the "Convenience" and "Anticoagulant Treatment Satisfaction" dimensions displayed good convergent and discriminant validity. The internal consistency reliability was good, with a Cronbach's alpha of 0.84 for the "Convenience" dimension, and 0.76 for the "Anticoagulant Treatment Satisfaction" dimension. Known-group validity was good, especially with regard to occurrence of thromboembolic events within 3 months from randomisation.

**Conclusion:**

The PACT-Q is a valid and reliable instrument that allows the assessment of patients' expectations and satisfaction regarding anticoagulant treatment, as well as their opinion about treatment convenience of use. Its two-part structure – assessment of expectations at baseline in the first part, and of convenience, burden and treatment satisfaction in the second – was validated and displays good and stable psychometric properties. These results are not sufficient to recommend the use of satisfaction as primary endpoint in clinical trials; further validation work is needed to support the interpretation of PACT-Q dimension scores. However, this first validation makes the PACT-Q an appropriate measure for use in clinical and pharmacoepidemiological research, as well as in real-life studies.

**Trial Registration:**

(ClinicalTrials.gov numbers, NCT00067093, NCT00062803 and NCT00070655).

## Background

Oral vitamin K antagonists (VKA) are effective and commonly used anticoagulant agents for the secondary prevention of venous thromboembolic disease and the prevention of systemic embolism in patients with atrial fibrillation [1]. However, because of patients' monitoring requirements, their inherent limitations in daily life (e.g. diet and activities) and the considerable inter-individual variability in pharmacodynamic effect, the burden of VKA on patients' daily life is highly significant, especially when given as long-term therapy [2-4]. Together with possible side effects such as bleedings, anticoagulant treatment may negatively affect patients' health-related quality of life (HRQoL) and treatment satisfaction, which in turn is likely to result in the decrease of the treatment effectiveness and ultimately in its failure [5-9]. Patients' satisfaction with treatment mainly depends on their previous experiences of similar treatment to which they compare their new treatment, as well as on their expectations [10]. Thus, especially when clinical outcomes regarding treatment efficacy and tolerance are rare and undistinguishable between different treatments, information about patients' satisfaction with their treatment and HRQoL is highly valuable. Surprisingly, despite the large use of VKA, no specific questionnaire was available to assess patients' satisfaction with their treatment and to evaluate their unmet needs. Therefore, the Perception of Anti-Coagulant Treatment Questionnaire (PACT-Q) was developed [11] as a means to investigate the burden of disease in patients with deep venous thrombosis (DVT), pulmonary embolism (PE) or atrial fibrillation (AF), with specific focus on patients' satisfaction with anticoagulant treatment and treatment convenience. The questionnaire was administered in the United States, the Netherlands and France during international phase III clinical trials in patients with DVT, PE or AF. These studies aimed at evaluating the efficacy and safety of the new anticoagulant drug -idraparinux- injected subcutaneously once a week, compared to VKA. The trials were multicentre, international, randomised in two arms (VKA versus idraparinux), open-label and assessor-blind.

In order to be fully acceptable as a meaningful endpoint in clinical trials, the structure of a questionnaire has to be validated, its scoring rules established and its psychometric properties demonstrated. In this paper, we present the finalisation, i.e. scoring and validation of the original structure of the questionnaire. The psychometric validation of the resulting dimensions is also presented.

## Methods

### The original PACT-Q

The original PACT-Q consists of two parts and contains 27 items [11]: the PACT-Q1, composed of a single dimension (7 items), covering the expectations of patients regarding their anticoagulant treatment, is to be administered before treatment initiation; the PACT-Q2, composed of 3 dimensions covering the convenience of use of the treatment (11 items), burden of disease and treatment (2 items) and satisfaction with the anticoagulant treatment (7 items) as perceived by the patient, is to be administered to patients once the treatment is ongoing [11]. All the items of PACT-Q1 and PACT-Q2 parts are to be answered on a 5-point Likert scale. The questionnaire was simultaneously developed in French, American English and Dutch following a rigorous development process, in which the data collected from the patients were given particular importance [11]. It was further translated and adapted into 11 different country-specific versions following recommended linguistic validation procedures that included forward and backward translations by native speakers [12].

### PACT-Q administration

The PACT-Q was administered during phase III clinical trials (DVT Clinical Study Protocol 64717/EFC3491; PE Clinical Study Protocol 64714/EFC 3484; AF Clinical Study Protocol 64720) as secondary endpoint in multicentre studies conducted in Europe (Austria, Belgium, Czech Republic, Denmark, France, Germany, Italy, the Netherlands and Poland), North America (Canada and the United States) and Oceania (Australia and New Zealand), with patients with DVT, AF or PE. All three trials were randomised, open-label and assessor-blind, and were conducted in accordance with the principles stated in the Declaration of Helsinki. Patients who provided an oral consent prior to their inclusion participated in the study. At Day 1, patients were asked to complete the PACT-Q1 part of the questionnaire; at 3 month (M3) and 6 month (M6), patients were asked to complete the PACT-Q2 part.

### Definition of the study populations

**Figure 1:**
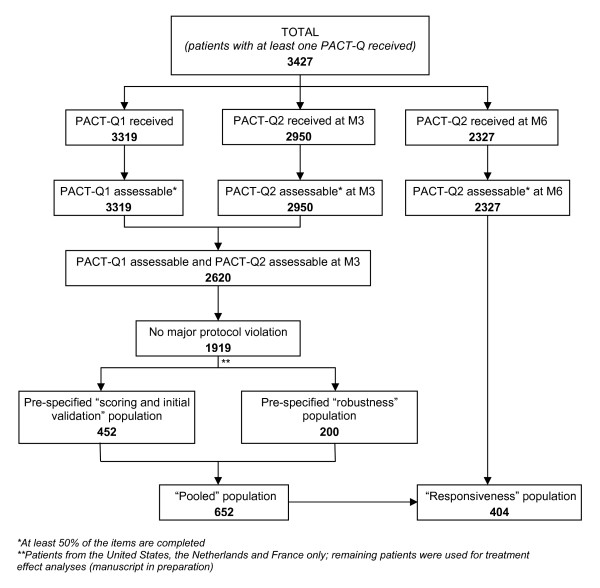
**Flowchart of the population included and participating in the study**.

Patients included in the analyses had at least one PACT-Q1 and one PACT-Q2 completed and assessable (i.e. 50% of items completed) at Day 1 and M3, respectively, and did not violate the protocol for PACT-Q administration along the whole study (Figure 1). As the PACT-Q was originally developed in French, American English and Dutch, only patients from France, the United States and the Netherlands were included in the study populations. In order to control learning bias and for the purpose of the validation [13], two populations were pre-specified: the "scoring and initial validation" population subset was constituted of 452 patients with either DVT, AF or PE and from either the United States, the Netherlands or France; the "robustness" population subset was constituted of 200 AF patients from either the United States or the Netherlands. The "pooled" population (n = 652), corresponding to the "scoring and initial validation" population subset combined with the "robustness" population subset, was used to determine the PACT-Q psychometric properties. Patients who answered at least 50% of the PACT-Q2 items at M6 were included in the responsiveness analysis of PACT-Q2 ("responsiveness" population subset).

### Statistical analyses

One should note that although the clinical trial was open-label, statistical analyses were blind to treatment attribution, i.e. no distinction was made between the treatment arms that patients were randomly allocated to.

#### Finalisation: scoring and initial validation of PACT-Q original structure

Principal Component Analysis (PCA) with Varimax Rotation is often used to assess the hypothetical structure of an instrument, by observing how the different items are spontaneously grouped into factors [14]. In our case, PCA was performed to check whether the factorial structure of PACT-Q reproduces the number and the type of dimensions that were hypothesised during the development step. Multitrait analysis (MA) defining the item convergent validity criterion (correlation between each item and its own dimension is considered satisfactory if it achieves ≥ 0.40) and the item discriminant validity criterion (each item should have a higher correlation with its own dimension than with the other dimensions) was performed to ascertain the consistency of the dimensions [15].

The internal consistency reliability of the dimensions [16], i.e. the extent to which individual items are consistent with each other and reflect a single underlying construct, was assessed by the determination of Cronbach's alpha coefficient [17]. A value of 0.70 or above is recommended for group comparisons [18]. A Multiple Factorial Analysis (MFA) was carried out to analyse the relationship between PACT-Q1 and PACT-Q2 [19]. MFA assesses several sets of variables defined on the same set of individuals, and allows computation of a summary statistic referred to as the RV coefficient (vector correlation coefficient) [20]. RV value ranges from 0 to 1; the closer to 1 the RV, the more similar the configuration of the two sets of variables.

#### Validation and assessment of the psychometric properties of the PACT-Q dimensions

Validity is the degree to which the instrument measures what it is supposed to measure [21-23]. Several types of validity were assessed. The validation of the conceptual model was assessed by performing MA with description of item convergent and discriminant validity [15]. Scale-scale correlation was evaluated in order to test the construct validity of the questionnaire. Floor and ceiling effects were measured to check that there was no issue related to a high percentage of patients having the lowest or the highest possible score on any one scale. Clinical validity evaluates the extent to which the questionnaire is able to detect variability amongst patients with different clinical severity levels. Known-group validity can be evaluated when differences in scores are expected between groups of patients that differ on relevant variables. Clinical and known-group properties were assessed amongst groups of patients defined according to age, gender, international normalized ratio (INR – patients at risk of embolism, INR < 2; patients with less risk of embolism, INR > 3), prior medication, thromboembolic events within the 3 months following randomisation, and time they spent in the 2–3 INR range between randomisation and M3.

Internal consistency reliability of the dimensions of the PACT-Q was again evaluated, by determination of the Cronbach's alpha coefficient [17]. Responsiveness refers to the ability of a questionnaire to detect important changes over a period of time [24]; it was assessed for the "Convenience" and "Anticoagulant Treatment Satisfaction" dimensions of the PACT-Q2 over M3 and M6, by determining the Effect-Size (ES) and Standardised Response Mean (SRM) [25-27]. A Wilcoxon signed rank test was performed in order to compare the change to 0. A Kruskal-Wallis test was performed to test the hypothesis that the change in scores was significantly different across the different groups of patients. The PACT-Q responsiveness was tested for groups of patients defined according to the primary efficacy outcome (i.e. thromboembolic event within 3 months from randomisation consisting in stroke or a non-central nervous system systemic embolism for AF patients, and a PE or DVT event for PE and DVT patients respectively) and the time spent in the 2–3 INR range between randomisation and M6 visits. The threshold for statistical significance was set at 0.05.

MFA was performed using SPAD Software. All the other data processing and analyses were performed with SAS Software for Windows (Statistical Analysis System, version 9).

## Results

### Population characteristics

Socio-demographic and clinical characteristics of the "pooled" population set that was used in the analyses are summarised in Table 1. The "pooled" population consisted of 652 patients, amongst whom 234 were from the United States, 309 from the Netherlands and 109 from France. Of these 652 patients, 426 patients had AF, 87 had DVT and 139 had PE. The mean age was 65 years, patients with AF being the oldest population (mean = 68 years-old). Overall, males were more represented than females, with the greatest difference observed within the AF population (70% men).

**Table 1:** Socio-demographic and clinical characteristics of the "pooled" population at day 1 (n = 652)

**Medical condition**	**Number of patients**	**Country^a ^(n)**			**Age** **(years ± SD)**	**Gender (%)**		**INR^b, c ^(n)**			**Patients with prior medication^d ^(n)**
		**US**	**NL**	**FR**		**Male**	**Female**	**<2**	**[2;3]**	**>3**	
**Atrial fibrillation**	426	152	251	23	68 ± 9.5	70	30	152	207	64	72
**Deep venous thrombosis**	87	58	0	29	54.6 ± 16.8	48	52	77	0	0	0
**Pulmonary embolism**	139	24	58	57	59.9 ± 16.4	52	48	11	1	0	0

^a ^US, The United States; NL, The Netherlands; FR, France

^b ^INR, International Normalized Ratio; MD

^c ^MD, missing data = 39

^d ^Patients who had already received antithrombotic agents prior to participation in the study

### Challenging the original PACT-Q structure

The analyses were performed with the "scoring and initial validation" population subset (n = 452), including patients with AF (n = 226), DVT (n = 87) and PE (n = 139) from France (n = 109), the Netherlands (n = 209) and the United States (n = 134).

#### Finalisation of the PACT-Q: initial validation of the original structure and scoring

The PCA with Varimax Rotation and MA were conducted on the PACT-Q1 completed at Day 1. The PCA performed on the 7 items of PACT-Q1 resulted in the definition of 2 factors with eigenvalues greater than 1, accounting for only 23% (Factor 1, containing items A3, A5 and A7) and 18% (Factor 2, containing items A1, A2, A4 and A6) of the total variance. Correlations between the 7 items were very low, with Pearson coefficients ranging from -0.068 to 0.268, indicating that the items were non-redundant. Cronbach's alpha was low (0.43). Item-dimension correlation coefficients ranged from 0.12 to 0.29, reflecting low item convergent validity criteria. Taken together, these data indicated that the "Treatment Expectations" dimension was not unidimensional. Each of the items of this dimension will therefore be analysed separately.

Following PCA analysis with Varimax Rotation conducted on the PACT-Q2 part completed at M3, four factors were retained based on an eigenvalue greater than one, representing 53% of the total variance. Factor 1 contained all items of the original "Convenience" dimension (i.e. B1 to B9), except B10 and B11; factor 2 contained items D4 to D7 of the original "Anticoagulant Treatment Satisfaction" dimension; factor 3 the items B10, B11 of the "Convenience" dimension and the items C1 and C2 of the "Burden of Disease and Treatment" dimension; factor 4 the items D1, D2 and D3 of the original "Anticoagulant Treatment Satisfaction" dimension. MA results showed good item convergent validity criteria for all the items within their own respective dimension, except items B10 and B11 ("Convenience" dimension) and items D2 and D3 ("Anticoagulant Treatment Satisfaction" dimension). All items reached the discriminant validity criterion, i.e. all items shared a higher correlation with their own dimension than with the other dimensions of the questionnaire. Cronbach's alpha was satisfactory for the "Burden of Disease and Treatment" dimension (0.66), and good for the "Anticoagulant Treatment Satisfaction" and the "Convenience" dimensions (0.79 and 0.82, respectively). No ceiling effects were reported, whereas a floor effect was observed for the "Burden of Disease and Treatment" (57%) and "Convenience" (26%) dimensions.

To improve the measurement model of the PACT-Q2, several alternative models were further tested for their structure, amongst which one model was retained as it displayed the most satisfactory and stable properties. The retained model included 2 dimensions ("Convenience", containing all B and C items, and "Anticoagulant Treatment Satisfaction" dimension, containing all D items). All the items within each of the PACT-Q2 dimensions met the convergent validity criterion (ranging from 0.42 to 0.66), except items B10 and B11, and items D2 and D3. All items met the item discriminant validity criterion. A floor effect was still observed for the "Convenience" dimension; no ceiling effect was noted for the 2 dimensions. The respective Cronbach's alpha values were 0.83 and 0.79 for the "Convenience" and "Anticoagulant Treatment Satisfaction" dimensions. The correlation coefficient between the 2 dimensions was low (-0.25), confirming that they clearly covered two separate concepts.

**Figure 2:**
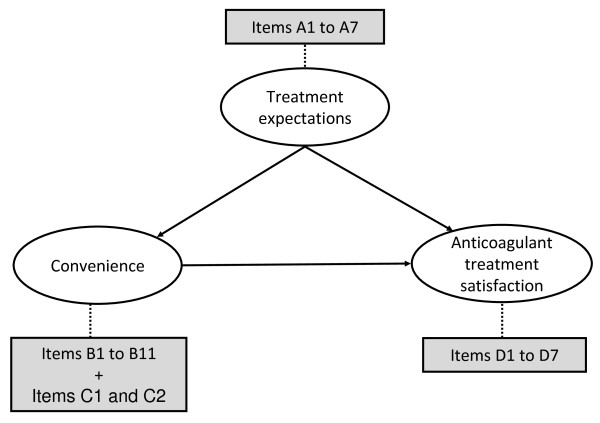
**Conceptual framework of the final PACT-Q**.

Thus, the final PACT-Q2 was constituted of 2 dimensions: the "Convenience" dimension, comprising items B1 to B11 (from the original "Convenience" dimension) combined with items C1 and C2 (from the original "Burden of Disease and Treatment" dimension), and the "Anticoagulant Treatment Satisfaction" dimension containing items D1 to D7 (Figure 2). The structure, dimensions and detailed contents of the items of the final version of PACT-Q are reported in Table 3.

The final PACT-Q1 remained similar to the original version, i.e. containing 7 items, each individually scored from 1 to 5; for items A1, A2, A4 and A5, the higher the score, the higher the patients' expectations of their treatment; for items A3, A5 and A7, the lower the score, the higher the patients' expectations of their treatment (Table 2). Within the PACT-Q2, items B and C are reversed, summed and rescaled on a 0–100 scale to obtain the "Convenience" dimension score (Table 2). The higher the score, the higher the convenience and the lower the burden as perceived by the patient for their treatment. Items D are summed and rescaled on a 0–100 scale to determine the "Anticoagulant Treatment Satisfaction" dimension score (Table 2); the higher the score, the higher the patient satisfaction with their anticoagulant treatment.

**Table 2:** Dimensions, detailed concepts and scoring method of the final PACT-Q

**PACT-Q**	**Dimensions**	**Item number – Detailed concept**	**Scoring**	**Score range**
**PACT-Q1**	**Treatment Expectations**	A1 – Confidence in prevention of blood clots	One score	1 to 5
		A2 – Expectations of symptom relief	One score	1 to 5
		A3 – Expectations of side effects	One score	1 to 5
		A4 – Importance of ease of use	One score	1 to 5
		A5 – Worries about making mistakes	One score	1 to 5
		A6 – Importance of independency	One score	1 to 5
		A7 – Worries about cost	One score	1 to 5
**PACT-Q2**	Convenience*	B1 – Difficulties in taking the treatment	One score	0 to 100
		B2 – Bother in taking the treatment		
		B3 – Difficulties regarding dose adjustments required		
		B4 – Treatment and other medications		
		B5 – Treatment and regimen implications		
		B6 – Treatment and being away from home		
		B7 – Difficulties regarding daily life		
		B8 – Bother in follow-up required		
		B9 – Difficulties regarding regular intake		
		B10 – Feeling regarding loss of independency		
		B11 – Worries about having to stop the treatment		
		C1 – Impact of side effects on usual activities		
		C2 – Discomfort due to symptoms		
	Anticoagulant Treatment Satisfaction*	D1 – Feeling of reassurance	One score	0 to 100
		D2 – Symptom decrease		
		D3 – Experience with side effects		
		D4 – Satisfaction regarding independency		
		D5 – Satisfaction with patient management		
		D6 – Satisfaction with treatment form		
		D7 – Overall satisfaction		

* For convenience score, all items are reversed (reversed item score = 6 – initial item score), then summed and rescaled on a 0 – 100 scale; for satisfaction score, all items are summed and rescaled on a 0 – 100 scale; for both scores, the higher the score, the higher the convenience/satisfaction

**Table 3:** Description of PACT-Q2 dimension scores at M3 and M6

	**PACT-Q2 dimensions**	**N**	**Mean (STD)**	**Median (Q1 – Q3)**	**Min – Max**
**M3**	**Convenience**	651	91.3 (10.4)	94.2 (88.5 – 98.1)	32.7 – 100.0
	**Anticoagulant Treatment Satisfaction**	641	68.9 (17.0)	67.9 (57.1 – 79.2)	0.0 – 100.0

**M6**	**Convenience**	404	90.6 (10.4)	94.0 (86.5 – 98.1)	23.1 – 100.0
	**Anticoagulant Treatment Satisfaction**	403	70.6 (17.4)	71.4 (60.7 – 82.1)	0.0 – 100.0

#### Studies of the link between PACT-Q1 and PACT-Q2

Pearson correlation coefficients between PACT-Q1 and PACT-Q2 (final structure) were below 0.20 for the majority of the items, indicating a weak relationship between the "Treatment Expectations" items and those assessing the "Convenience" and "Anticoagulant Treatment Satisfaction". RV coefficients between PACT-Q1 at Day 1 and PACT-Q2 at M3 were also low, ranging from 0.032 to 0.040, showing weak links between the dimensions of the two PACT-Q parts.

### Validation and psychometric properties of the final PACT-Q dimensions

MA was first conducted with the "scoring and initial validation" population subset (n = 452). In order to use a subset of patients that had not been used for the scoring, the robustness of the PACT-Q structure was then validated with the "robustness" population subset (n = 200). A further analysis with the "pooled" population set (n = 652) allowed the validation to be consolidated.

Regardless of the country, the quality of completion of PACT-Q1 at Day 1 was good in the "pooled" population, with 1.1% missing data. For PACT-Q2, completion was still good though slightly lower, with 2.4% missing data at M3, and 1.5% missing data at M6.

**Figure 3:**
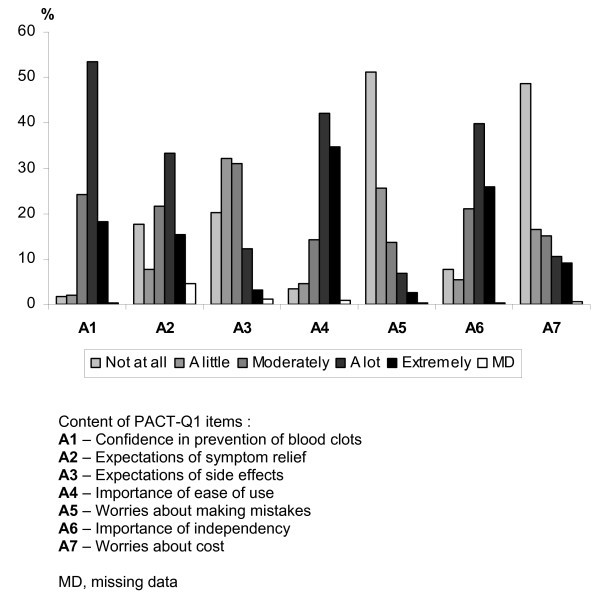
**Description of the percentage of patients per response choice to the "Expectations" items at Day 1 (N = 652)**.

Percentages of responses allocated to each of the response choices at Day 1 for the "Expectations" items are represented on Figure 3. The majority of patients answered "A lot" or "Extremely" for items A1, A2, A4 and A6. The majority of patients answered "Not at all" or "A little" for items A3, A5 and A7. Scores of the "Convenience" and "Anticoagulant Treatment Satisfaction" at M3 and M6 are summarised in Table 4. "Convenience" scores decreased from 91.3 at M3 to 90.6 at M6; "Anticoagulant Treatment Satisfaction" scores slightly increased from 68.9 to 70.6, respectively.

**Table 4:** Psychometric validation of the PACT-Q2 dimensions for the 3 population subsets

**Population subsets**	**Cronbach's alpha**	**Floor effect^c ^(%)**	**Ceiling effect^d ^(%)**	**Convergent and discriminant validity criterion** **(range of item-scale correlations)**
***"scoring and initial validation" (n = 452)***				
**Convenience**	0.83	0	21.2	0.31–0.63
**Anticoagulant Treatment Satisfaction**	0.79	0.6	4.0	0.36–0.66

***"robustness" (n = 200)***				
**Convenience**	0.87	0	24.1	0.40–0.67
**Anticoagulant Treatment Satisfaction**	0.70	0	1.9	0.26–0.59

***"pooled" (n = 652)***				
**Convenience**	0.84	0	22.1	0.33–0.64^a^
**Anticoagulant Treatment Satisfaction**	0.76	0.4	3.3	0.33–0.63^b^

^a ^except items B10 and B11, all items meet the convergent validity criterion

^b ^except items D2 and D3, all items meet the convergent validity criterion

^c ^percentage of patients with the lowest possible score (i.e. 0), that is patients who answered the least favourable response choice to all items of the dimension.

^d ^percentage of patients with the highest possible score (i.e. 100), that is patients who answered the most favourable response choice to all items of the dimension.

Item convergent validity, floor and ceiling effects and internal consistency reliability of the PACT-Q2 dimensions obtained for these two subsets of population and the "pooled" population are summarised in Table 4.

#### Item convergent and discriminant validity criteria

Overall (i.e. in the "pooled" population), all the items within the "Convenience" dimension, except items B10 and B11, met the item convergent criteria and ranged from 0.33 to 0.64. All the items within the "Anticoagulant Treatment Satisfaction" dimension met the convergent criterion, except items D2 and D3, and ranged from 0.33 to 0.63. All the items of the 2 dimensions reached the item discriminant validity criterion.

#### Scale-scale correlation

The correlation coefficient between the "Convenience" and "Anticoagulant Treatment Satisfaction" dimensions of the PACT-Q2 was moderate (0.29), reflecting a fair relationship and no redundancy between the 2 dimensions.

#### Floor and ceiling effects

No floor effect was observed for either dimension of the PACT-Q2. No ceiling effect was noted for the "Anticoagulant Treatment Satisfaction" dimension, contrary to the "Convenience" dimension (percentage of patients at the highest possible score = 22%).

#### Internal consistency reliability

Good internal consistency reliability was displayed within each of the PACT-Q2 dimensions, with Cronbach's alpha values of 0.84 for "Convenience", and 0.76 for "Anticoagulant Treatment Satisfaction".

#### Known-group validity

Scores of items A2, A4, A5 and A7 within the "Treatment Expectations" dimension at Day 1 (PACT-Q1), and the "Convenience" and "Anticoagulant Treatment Satisfaction" dimensions at M3 showed significant differences when compared according to the age of the patients: the highest scores of "Treatment Expectations" items were observed for patients under 60 years of age (data not shown). Patients aged between 60 and 65 displayed higher "Anticoagulant Treatment Satisfaction" scores than younger and older patients, this difference being significant (p = 0.028). For the "Convenience" dimension, the highest score was observed for patients aged between 65 and 75 years (p < 0.0001). Only the A2, A3 and A7 item scores of "Treatment Expectations" were significantly different between females and males, while other scores were similar over the 2 groups. When compared according to experience of prior medication, scores of the "Convenience" dimension were significantly higher for patients who did not have prior medication than patients who did (score of 92 versus 89, respectively; p = 0.01). A higher score was reported at item A2 ("Treatment Expectations" – symptom relief) for patients with no prior medication experience than for patients with experience (3.3 versus 2.8, p = 0.0044). In contrast, a significantly higher score for item A3 ("Treatment Expectations" – side effects) was observed for patients with prior experience of medication than patients with no prior medication (2.9 versus 2.4, p = 0.0003). Scores in the "Anticoagulant Treatment Satisfaction" dimension were higher for patients who reported no events within 3 months of randomisation than for those who reported having had an event (69 versus 41, respectively; p = 0.005).

When compared between groups of patients defined according to their baseline INR level at Day 1 (i.e. < 2, [2;3], > 3), scores of most items of PACT-Q1 (item A2, "symptom relief"; item A3, "side effects"; A4, "ease of use"; A5, "making mistakes"; and A7, "cost of the treatment") were significantly higher for patients with INR < 2 than for patients with INR = 2. Regarding PACT-Q2 at M3, the "Anticoagulant Treatment Satisfaction" score was the lowest for patients with an INR > 3, and significantly increased as INR decreased (scores ranging from 61 to 69). These results at M3 concerned only patients treated with VKA as the INR was not available after Day 1 for patients treated with the new anticoagulant treatment. None of the other differences observed between scores of the PACT-Q2 dimensions or PACT-Q1 items were significant.

Similarly, score differences reported between groups of patients defined according to the time they spent in the 2–3 INR range (4 groups defined: < 50%, between 50% and 60%, between 60% and 70%, and ≥ 70%) were not significant. In other terms, the time spent in the 2–3 INR range between randomisation and M3 does not have a statistically significant impact on scores. Again, these results concerned only patients treated with VKA, as those treated with idraparinux did not have INR monitoring during the study.

#### Change of PACT-Q2 scores over 3 months

Overall, no significantly different changes in the "Convenience" and "Anticoagulant Treatment Satisfaction" scores were observed from M3 to M6, whether compared between groups of patients defined according to their report of a thromboembolic event (or not) within 3 months of randomisation, the time they spent within the 2–3 INR range between randomisation and M3, and M3 and M6, or the impact of the INR control status deterioration or improvement. Most of the changes in scores observed within each group were not significantly different to 0 (p-signed rank test > 0.05).

## Discussion

The PACT-Q was included as a secondary endpoint in phase III clinical trials in order to assess the expectations and satisfaction of patients suffering from DVT, PE or AF and treated with a new long-acting anti-thrombotic drug injected once a week subcutaneously, in comparison with an oral VKA treatment. The finalisation and psychometric validation steps of the questionnaire were performed using the patients of the clinical trials.

### Validity of the original PACT-Q structure

Regarding the PACT-Q2 part, the exploratory PCA performed on the original PACT-Q revealed a structure very close to the proposed original structure [11], with 4 factors covering treatment convenience (items B1 to B9), treatment satisfaction (items D4 to D7), burden of disease and treatment and convenience of use (items C1 and C2 and items B10 and B11, respectively), and patients' anticoagulant treatment satisfaction (items D1 to D3). The overall good homogeneity of the PACT-Q2 dimensions, as reflected by the Cronbach's alpha values of 0.79 ("Anticoagulant Treatment Satisfaction" dimension) and 0.82 ("Convenience" dimension), confirmed the well-founded hypothesis of the original structure of the questionnaire. The lower value (0.66) reported for the "Burden of Disease and Treatment" dimension is perfectly acceptable as it contains only 2 items. As for the PACT-Q1 part, the weak correlations between each of the items and their low item convergent validity criterion, together with the low internal consistency reliability of the "Treatment Expectations" dimension, strongly suggests that the items of PACT-Q1 cannot be thought of as one single concept.

### Scoring and psychometric validation of the PACT-Q

The last series of PCA and MA led to the conclusion that the 7 items of the "Treatment Expectations" of PACT-Q1 were to be scored individually. This is not surprising as expectations cover heterogeneous concepts, each of them being measured by a single item. After the last finalisation step, the PACT-Q2 was reduced from 3 to 2 dimensions covering treatment "Convenience" (13 items) and "Anticoagulant Treatment Satisfaction" (7 items). The number of items in the 2 dimensions is quite high, and one could wish to reduce the PACT-Q to a short form. However, no items were removed as they were all relevant for the purpose of the questionnaire and the concepts were shown to be pertinent and well-accepted by patients. The use of PACT-Q in further clinical studies would be needed to definitively confirm their relevance. The reliability of these findings is increased by the use of separate subsets of population for the scoring and initial validation and for the psychometric validation.

The good quality of completion and returns of both PACT-Q1 and PACT-Q2 in all of the countries reflected the good acceptability of the questionnaire. The PACT-Q2 structure was further confirmed by a good item convergent and discriminant validity criterion and excellent internal consistency reliability of both dimensions. No floor or ceiling effects were observed for the "Anticoagulant Treatment Satisfaction" dimension; in contrast, a ceiling effect (22%) was reported for the "Convenience" dimension. While a ceiling effect is not recommended when measuring quality of life aspects [21], it is easily conceivable and acceptable that a majority of patients report no inconvenience of use in their treatment, particularly in clinical trials.

Known-group validity showed that PACT-Q dimensions were able to discriminate between groups of patients presenting different disease experiences and characteristics. In particular, patients who had had no previous experience of anticoagulant treatment were significantly more demanding in terms of symptom relief, while patients who had already been treated with anticoagulants expected more side effects than those who had not, and could therefore possibly be more realistic. These findings agree with Oliver's work, who proposed that patients' expectations are considerably influenced by previous experiences and knowledge that they have acquired of their disease and treatment [10]. As expected, patients' satisfaction was greater if they did not have a thrombolic event within the period of time that they were under treatment. In addition, one should point out that although a few patients only (n = 4) experienced an event within these 3 months, this had a noticeable impact on their satisfaction, thus indicating that PACT-Q is sensitive to events that are meaningful to patients. Lastly, patients at risk of embolism (INR < 2) at Day 1 of the study expected much more from their anticoagulant treatment in terms of cost, ease of use, side effects, worries about making mistakes and symptom relief, when compared to patients with lower risk of embolism (INR > 3). After 3 months of anticoagulant treatment, patients with an INR > 3 (i.e. with less risk of embolism) were the most satisfied with their treatment compared to patients with a higher INR. Interestingly, no correlation could be drawn between patients' expectations from the anticoagulant treatment at baseline and their satisfaction after 3 months of treatment. This can be explained by the theoretical model of Oliver et al., in which the authors propose that patients derive their satisfaction from the comparison between their expectations before starting the treatment and the performance they eventually perceive from it after being treated [10]. Therefore, in order to study the link between expectations and satisfaction, it would be necessary to assess it according to the treatment patients are receiving, which could not be performed as the statistical analyses were blind to the treatment attribution.

The good psychometric properties demonstrated by the PACT-Q in this paper support its use as secondary endpoint in trials. However, one should note that key validation criteria still need to be assessed before considering its use as a primary endpoint; in particular, the assessment of the responsiveness and test-retest reliability properties of the questionnaire and the estimation of the value for the minimal important difference.

Given the design of the phase III clinical trial, test-retest reliability of the PACT-Q was not assessed. For similar reasons, it was difficult to assess the responsiveness of the questionnaire. Indeed, as the PACT-Q2 aims to assess patients' satisfaction with their treatment and treatment convenience, the largest difference that one could expect should be between patients treated with VKA and patients treated with the new anticoagulant drug. However, the scoring procedure had to be established blinded to treatment groups; comparison of patients' satisfaction between these groups could therefore not be made in this present study. Changes in satisfaction and treatment convenience, are more likely to be assessable from the treatment effect longitudinal study; these data of which will be further presented in another publication.

## Conclusion

The PACT-Q is a valid and robust instrument that allows patients' expectations and satisfaction with anticoagulant treatment to be assessed. The good acceptability and psychometric properties of the questionnaire have been demonstrated with patients with various conditions including DVT, PE and AF, suggesting the PACT-Q's suitability for thromboembolic pathologies in general. Its multi-language validated versions will facilitate and widen its use in international studies.

This instrument could be helpful for clinicians to identify patients' expectations, either positive or negative, of their anticoagulant treatment. Together with a better knowledge of how patients perceive their treatment, the questionnaire could therefore contribute to facilitate physicians' decision about the most appropriate medical care for their patients; this would probably result in a better compliance from patients, and ultimately in a better control of the disease.

## Competing interests

The present work was funded by Sanofi-Aventis, Research and Development. The phase III trials were initiated by Sanofi-Aventis, Research and Development. The work on the PACT-Q was investigator-initiated. SR Kahn is supported by a Senior Clinical Investigator Award from the Fonds de la Recherche en Santé du Québec. Hélène Gilet, Isabelle Guillemin are paid consultants to Sanofi-Aventis, Research and Development. Sylvie Gabriel is an employee of Sanofi-Aventis, Research and Development. The other authors have no conflict of interest.

## Authors' contributions

All authors provided intellectual contributions to this manuscript. MHP participated in the conception and design of the questionnaire, and in the data interpretation. IG participated in the data interpretation and was responsible for writing the manuscript. HG was responsible for statistical analyses and participated in the data interpretation. SG participated in the conception and the design of the questionnaire. BE provided input on the questionnaire development and data interpretation. GR contributed to the questionnaire conception and design and data interpretation. SRK provided input on the questionnaire development and data interpretation

## Copyrights

PACT-Q^© ^is protected by copyright with all rights reserved to Sanofi-Aventis, France. Do not use without permission. For information on, or permission to use PACT-Q^©^, please contact the Mapi Research Trust, 27 rue de la Villette 69003 Lyon, France. Tel: +33 (0)4 72 13 65 75 – E-mail: trust@mapi.fr – website: .
